# Advanced Glycation End Product Inhibitor Pyridoxamine Attenuates IVD Degeneration in Type 2 Diabetic Rats

**DOI:** 10.3390/ijms21249709

**Published:** 2020-12-19

**Authors:** Juliane D. Glaeser, Derek Ju, Wafa Tawackoli, Jae H. Yang, Khosrowdad Salehi, Tina Stefanovic, Linda E. A. Kanim, Pablo Avalos, Giselle Kaneda, Stephen Stephan, Melodie F. Metzger, Hyun W. Bae, Dmitriy Sheyn

**Affiliations:** 1 Orthopaedic Stem Cell Research Laboratory, Cedars-Sinai Medical Center, Los Angeles, CA 90048, USA; juliane.glaeser@cshs.org (J.D.G.); derekju12@gmail.com (D.J.); Wafa.Tawackoli@csmc.edu (W.T.); kuspine@naver.com (J.H.Y.); Khosrowdad.Salehi@cshs.org (K.S.); tina.stefanovic95@gmail.com (T.S.); Linda.Kanim@cshs.org (L.E.A.K.); Giselle.Kaneda@cshs.org (G.K.); Stephen.Stephan@cshs.org (S.S.); Hyun.Bae@cshs.org (H.W.B.); 2 Board of Governors Regenerative Medicine Institute, Cedars-Sinai Medical Center, Los Angeles, CA 90048, USA; Pablo.Avalos@cshs.org; 3 Department of Orthopedics, Cedars-Sinai Medical Center, Los Angeles, CA 90048, USA; 4 Department of Surgery, Cedars-Sinai Medical Center, Los Angeles, CA 90048, USA; 5 Biomedical Imaging Research Institute, Cedars-Sinai Medical Center, Los Angeles, CA 90048, USA; 6 Department of Biomedical Sciences, Cedars-Sinai Medical Center, Los Angeles, CA 90048, USA; 7 Korea University Guro Hospital, Seoul 08308, Korea; 8 The Orthopaedic Biomechanics Laboratory, Cedars-Sinai Medical Center, Los Angeles, CA 90048, USA; Melodie.Metzger@cshs.org

**Keywords:** intervertebral disc degeneration, type 2 diabetes mellitus, advanced glycation end products

## Abstract

Type 2 diabetes mellitus (T2DM) is associated with advanced glycation end product (AGE) enrichment and considered a risk factor for intervertebral disc (IVD) degeneration. We hypothesized that systemic AGE inhibition, achieved using pyridoxamine (PM), attenuates IVD degeneration in T2DM rats. To induce IVD degeneration, lumbar disc injury or sham surgery was performed on Zucker Diabetic Sprague Dawley (ZDSD) or control Sprague Dawley (SD) rats. Post-surgery, IVD-injured ZDSD rats received daily PM dissolved in drinking water or water only. The resulting groups were SD uninjured, SD injured, ZDSD uninjured, ZDSD injured, and ZDSD injured + PM. Levels of blood glycation and disc degeneration were investigated. At week 8 post-surgery, glycated serum protein (GSP) levels were increased in ZDSDs compared to SDs. PM treatment attenuated this increase. Micro-MRI analysis demonstrated IVD dehydration in injured versus uninjured SDs and ZDSDs. In the ZDSD injured + PM group, IVD dehydration was diminished compared to ZDSD injured. AGE levels were decreased and aggrecan levels increased in ZDSD injured + PM versus ZDSD injured rats. Histological and immunohistochemical analyses further supported the beneficial effect of PM. In summary, PM attenuated GSP levels and IVD degeneration processes in ZDSD rats, demonstrating its potential to attenuate IVD degeneration in addition to managing glycemia in T2DM.

## 1. Introduction

Low back pain (LBP) is a leading cause of global disability [1]. Previous research demonstrates an association between intervertebral disc (IVD) degeneration and LBP that increases as the number of affected intervertebral disc levels increases [2].

IVD degeneration is a multifactorial disease that includes alterations in function and the increased death of nucleus pulposus (NP) cells, upregulated levels of proinflammatory cytokines and associated catabolic enzymes in the disc microenvironment, and changes in biomechanical properties [3,4]. As an avascular tissue, the IVD has a very limited intrinsic healing potential [5]. Experimental strategies for the attenuation and regeneration of disc degeneration include the injection of anti-inflammatory agents, growth factors to stimulate extracellular matrix production and cell proliferation, and stem cell therapies [6,7,8,9]. 

Pre-clinical analyses of IVD degeneration use rabbit and rodent models, as well as porcine, ovine, caprine, and canine models [10]. In this study we used a rat model, since it is large enough for surgical alteration of the spinal structures and allows defined biobehavioral testing for analyzing discogenic pain [11]. In a prior study by our group, an 18-gauge (G) needle-induced IVD injury in rats led to consistent severe disc degeneration and was associated with discogenic pain [12].

Type 2 diabetes mellitus (T2DM) is characterized by insulin insensitivity as a result of insulin resistance, declining insulin production, and eventual pancreatic beta-cell failure, which leads to a decrease in glucose transport into the liver, muscle cells, and fat cells [13]. It is estimated that 366 million people worldwide had diabetes mellitus in 2011; by 2030 this number is projected to rise to 552 million [13].

Diabetes is associated with an accumulation of advanced glycation end products (AGEs) in human and animal tissues, which results in increased levels of inflammation [14]. The Maillard hypothesis proposes that these complications develop as a result of chronic increased chemical modification of tissue proteins by glucose during hyperglycemia [15]. During the initial, intermediate, and final stages of the Maillard reaction, different AGE compounds are created [16]. These include Nε-(carboxymethyl)lysine (CML), Nε-(carboxyethyl)lysine (CEL) and pyrraline, and intra- and inter-molecular crosslinks, such as pentosidine, glucosepane, and imidazolium compounds [17,18].

T2DM has been identified as a clinical risk factor for spinal diseases including IVD degeneration and disc herniation [19]. Human cohort studies demonstrate an increased incidence of diabetes among patients undergoing surgery for both cervical disc disease and lumbar disc disease, suggesting a possible predisposing factor for symptomatic disc disease [20]. The mechanism by which diabetes affects IVD degeneration is currently not well understood. Research findings indicate that the formation of AGEs in the NP is crucially involved in the progression of disc degeneration in T2DM [21].

In the IVD, AGEs accumulate in major proteins, including in aggrecan and collagen, which alters NP cell biology, preventing repair and turnover [22]. This results in a more fibrous, dehydrated matrix that is less equipped to withstand the mechanical forces typical of the spine. Therefore, increased AGE accumulation is hypothesized to accelerate the degenerative process of the diabetic NP [23].

Inhibition of AGE formation may prevent AGE accumulation and mitigate its associated effects on accelerated IVD degeneration and spine-related pain [24]. Supplementation with the vitamin B6 form, pyridoxamine (PM), has been demonstrated to reduce pathological changes to IVD structure and composition in diabetic mice [25], and diabetic neuropathy via suppression of a spinal receptor in a rat animal model [26]. Phase 2 clinical studies have reported promising results of daily PM use in patients with T2DM and nephropathy [27]. Similarly, the protective effects of PM in combination with anti-inflammatory treatment using pentosan-polysulfate on IVDs have been reported in a diabetic mouse model [25].

Various animal models have been employed to study T2DM [28,29]. The Zucker Diabetic Sprague-Dawley (ZDSD) animal model is a favorable model because it displays the gradual development of diabetes, mimicking the pathogenesis of human T2DM [30]. The ZDSD rat is a cross between homozygous lean Zucker Diabetic Fatty rats (ZDFfa/+) and a sub-strain of the Sprague-Dawley rat that was selectively bred for diet-induced obesity [31]. This model was previously established in a study by our group to assess the impact of diabetes on bone metabolism after spinal fusion [32].

In the present study, we hypothesized that systemic AGE inhibition attenuates injury-induced IVD degeneration in ZDSD rats. The first aim was to investigate differences in blood glycation, disc hydration, and morphology, as well as AGE and aggrecan regulation in diabetic and non-diabetic rats with degenerated IVDs. The second aim was to assess whether oral anti-AGE treatment may mitigate these diabetes-induced degenerative changes in the spine.

## 2. Results

### 2.1. PM Treatment Results in Reduction of Glycated Serum Protein Levels in IVD-Injured ZDSD Rats

**Figure 1:**
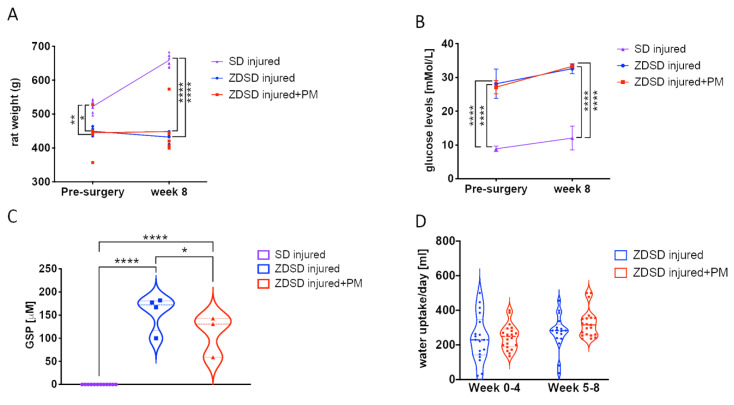
PM treatment results in reduction of GSP levels in ZDSD rats with injured IVDs. (**A**) Rat weight measurements (*n* ≥ 5), (**B**) blood glucose testing *(n* ≥ 4), (**C**) the detection of GSP (*n* ≥ 5) and (**D**) daily water uptake of the various experimental groups at different time points (*n* ≥ 5). GSP: glycated serum protein. * *p* < 0.05, ** *p* < 0.01, **** *p* < 0.0001.

Prior to surgery and at 8-weeks post-surgery, rat weight and glucose levels were evaluated in both male SD and ZDSD rats 20 weeks of age at the time of surgery. In the ZDSD injured + PM group, PM treatment (200 mg/kg/day) was given on a daily basis for 8 weeks, starting at day 1 post-surgery. At week 0, SD rats were on average heavier than ZDSD animals (SD: 523 ± 13 g; ZDSD: 450 ± 13 g, *p* < 0.05). At week 8, the mean SD rat weight further increased to 659 ± 17 g, while no significant change in the ZDSD rat weight was detected (Figure 1A, *p* < 0.0001). At the time of IVD needle injury, all ZDSD rats were confirmed to be diabetic based on random collections of plasma glucose levels exceeding 200 mg/dL (11.1 mmol/L) [33]. Blood glucose levels were increased in ZDSD rats versus (vs.) SD rats at weeks 0 and week 8 (Figure 1B, *p* < 0.0001). At weeks 0 and 8, no difference between rat weight and glucose levels in ZDSD and ZDSD + PM rats were detected (Figure 1A,B). Glycated serum protein (GSP) levels measured at week 8 were higher in the ZDSD and ZDSD + PM groups than in the SD group (*p* < 0.0001). In the ZDSD + PM group, GSP levels were lower than those found in the ZDSD group (Figure 1C, *p* < 0.05). No differences in daily water uptake were detected between ZDSD and ZDSD + PM rats (Figure 1D).

### 2.2. Oral Treatment with the AGE Inhibitor PM Attenuates NP Dehydration after Needle Injury

**Figure 2:**
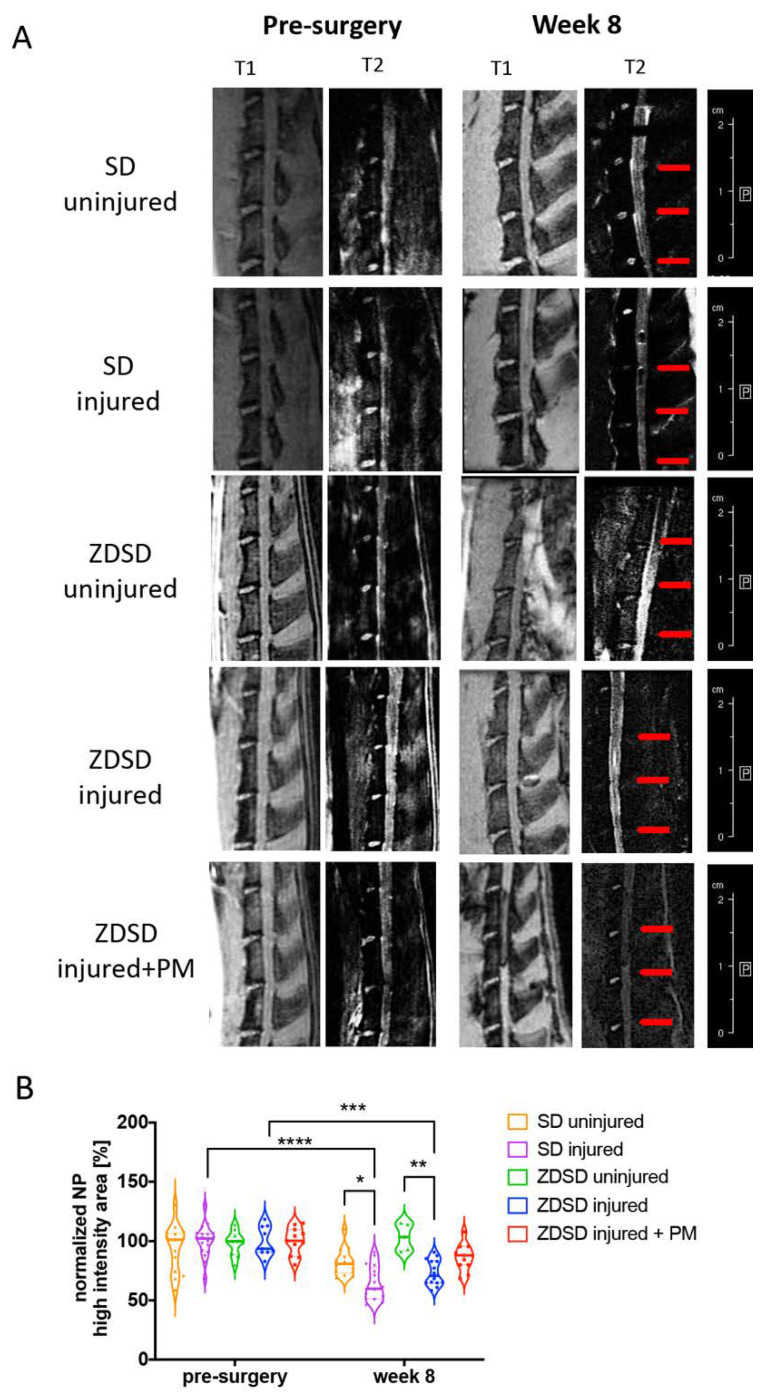
PM attenuates NP dehydration in needle-injured IVDs. (**A**) Representative images of IVD degeneration at week 8 vs. pre-surgery. Left: (T1) scans (TR: 50 ms, TE: 1.7 ms), right: T2-weighted scans (TR: 5000 ms, TE: 30 ms), obtained using Bruker BioSpec 9.4 T (94/20) with Avance III electronics 9.4 T. Red arrows indicate needle-injured IVDs. (**B**) Quantitative analysis of the µMRI images showing relative NP high-intensity areas (T2) in discs in the various SD and ZDSD rat groups pre-surgery and at 8 weeks post-surgery. µMRI data were normalized to mean uninjured discs at the same level from rats undergoing sham surgery. * *p* < 0.05, ** *p* < 0.01, *** *p* < 0.001, **** *p* < 0.0001, *n* ≥ 5.

T2-weighted μMRI image analysis demonstrated a reduction in NP hydration in the SD injured and ZDSD injured groups at 8 post-surgery compared to the same groups pre-surgery (SD injured pre-surgery vs. week 8 post-surgery: *p* < 0.0001; ZDSD injured pre-surgery vs. week 8 post-surgery: *p* < 0.001). At 8 weeks post-surgery, NP hydration was lower in the SD injured group than in the SD uninjured (*p* < 0.05) and lower in the ZDSD injured group than in the ZDSD uninjured group (*p* < 0.001). No significant difference in NP hydration was detected between ZDSD uninjured and ZDSD injured + PM (Figure 2A,B).

### 2.3. IVD Lesion and NP Destruction in Response to Needle Injury is Attenuated in Rats Receiving PM

**Figure 3:**
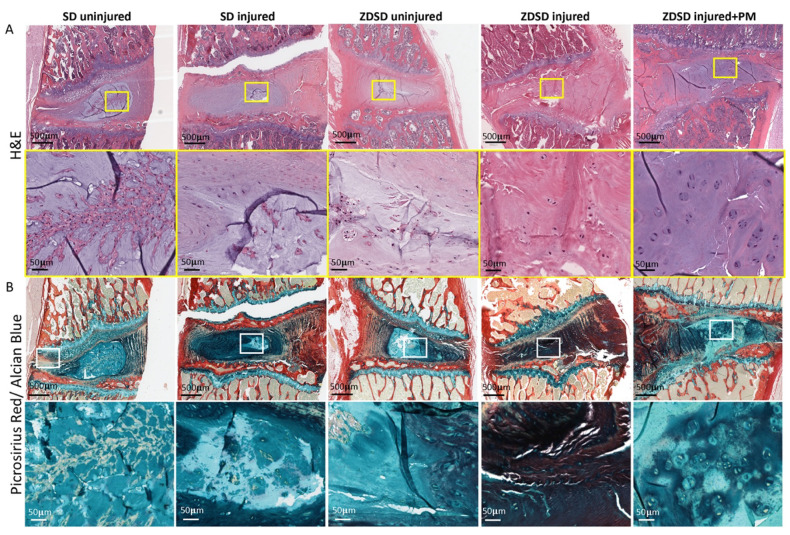
IVD lesion and NP destruction in response to needle injury is attenuated in rats receiving PM. (**A**) H&E-stained and (**B**) Picrosirius red/Alcian blue—stained lumbar discs from IVDs from various experimental groups harvested at 8 weeks post-surgery. Upper images in panels A and B show IVDs and adjacent endplates (scale bar: 500 μm). Lower images in panels A and B show magnification of the NP area (scale bar: 50 μm). Yellow and white rectangles indicate the area used for magnification, *n* ≥ 3.

Histological analysis including H&E and Picrosirius red/Alcian blue staining of spine segments obtained from the various experimental groups was performed. In the SD and ZDSD uninjured groups, the NP and the NP/AF interphase were fully intact. Compared to the SD uninjured group, the amount of small cell clusters surrounded by cell-free matrix was reduced in the ZDSD uninjured group. In the SD injured group, the NP was largely degenerated, with small cell/matrix islands remaining. In the ZDSD injured group, the complete destruction of NP tissue with no visible remaining NP and annulus fibrosus (AF) interphase was detected. In the ZDSD injured + PM group, the NP was partially destructed; however, intact cells were still present within the NP. Morphologically, the most severe degeneration was found in the ZDSD injured group (Figure 3A,B).

### 2.4. PM Treatment Decreases AGE Levels and Increases Aggrecan Levels in Needle-Injured IVDs

**Figure 4:**
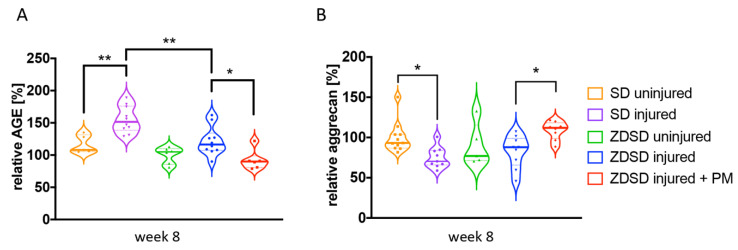
PM treatment decreases AGE levels and increases aggrecan levels in needle-injured IVDs. (**A**) Relative levels of AGE protein and (**B**) relative levels of aggrecan accumulation in homogenized IVD samples normalized to the levels detected in the ZDSD uninjured group. * *p* < 0.05, ** *p* < 0.01.

AGE and aggrecan protein levels in IVD tissue extracts from the various treatment groups were determined by ELISA. In SD rats, AGE levels were increased and aggrecan levels decreased in SD injured versus SD uninjured groups (AGE: *p* < 0.01; aggrecan: *p* < 0.05, Figure 4A,B). AGE levels were higher in the SD injured group than in the ZDSD injured group (*p* < 0.05, Figure 4A). AGE levels were decreased and aggrecan levels increased in the ZDSD injured + PM group compared with the ZDSD injured group (*p* < 0.05, Figure 4A,B). No differences in AGE and aggrecan levels were detected between the ZDSD uninjured and ZDSD injured groups.

### 2.5. PM Treatment Reduces AGE Levels in Injured IVDs and Adjacent Endplate Fibrocartilage

**Figure 5:**
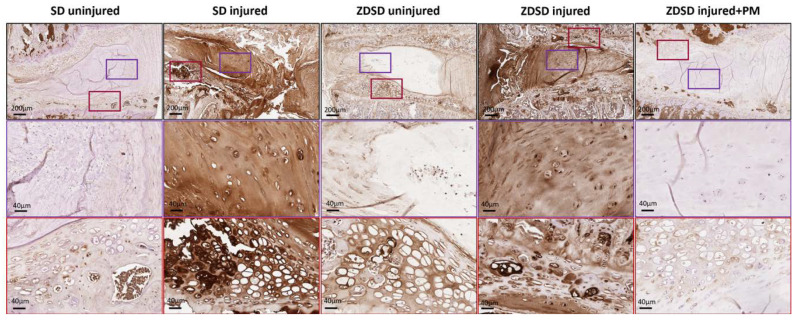
PM treatment reduces AGE levels in injured IVDs and adjacent endplate fibrocartilage. Immunohistochemical AGE staining of IVDs obtained from the various experimental groups harvested at 8 weeks post-surgery. Brown shading indicates positive AGE staining. Upper images show IVDs and adjacent endplates (scale bar: 200 μm). Middle images show magnification of the NP area (in purple rectangles; scale bar: 40 μm). Lower images show magnification of the fibrocartilage of the endplates (red rectangles; scale bar: 40 μm), *n* ≥ 3.

Using an antibody against a broad spectrum of AGE compounds, immunohistochemical staining of IVDs from the various experimental groups indicated increased AGE accumulation in the NP and, especially, in the adjacent fibrocartilage of endplates of uninjured IVDs from ZDSD rats compared to uninjured SD controls. AGE levels strongly increased in the NP and endplate fibrocartilage of both ZDSD and SD rats following needle injury. Oral PM treatment resulted in a reduction in AGE accumulation in the injured IVDs of ZDSD rats (Figure 5).

## 3. Discussion

The purpose of this study was to understand the interaction between type 2 diabetes and IVD degeneration, both of which cause substantial disability and financial costs to our society. In addition, the effect of systemic AGE inhibition on IVD degeneration was evaluated in a diabetes model. Results demonstrate increased levels of AGE compounds in the IVD and in the endplate fibrocartilage of uninjured IVDs of ZDSD rats versus those in SD rats. IVD needle injury resulted in NP dehydration, increased AGE accumulation, and decreased aggrecan levels in both ZDSD and SD rats. AGE inhibition achieved by PM treatment reduced GSP levels and attenuated IVD degeneration in ZDSD rats with injured IVDs, as demonstrated by µMRI and histology, reduced AGE levels in the NP and the endplate fibrocartilage, and increased aggrecan levels within the IVD.

SD rats weighed more than ZDSD rats regardless of whether the ZDSD animals received PM treatment (Figure 1A). At the beginning of the study, the control SD rats were slightly heavier than the experimental ZDSD rats. The SD rat weights further increased by week 8, while the ZDSD rat weights remained stable. In a prior study, no significant differences between ZDSD rats and non-diabetic CD (SD) rats were detected [34]. However, a different sub-strain may have been used in that study compared to the present one. In another study, the ZDSD model was demonstrated to gain weight following its high-fat diet until the onset of diabetes, after which the catabolic state of diabetes caused weight loss [35]. The advantage of lack of rapid weight gain in ZDSD rats is that it allows avoiding weight-related variables that could affect the mechanical properties of the IVD or spinal column. However, the differences in weight gain between ZDSD and SD rats in this study needs to be considered when comparing these two models with each other. Although the CD IGS SD rats used in our model were fed a regular diet, they weighed 659 ± 17 g at 28 weeks of age. This is just slightly less than the average weight detected in diet-induced obese CD IGS SD rats at 28 weeks (with an average weight of 680 g) [36]. Since we did not perform any obesity testing, such as fat mass determination, we cannot exclude that our SD rats developed obesity.

All ZDSD rats demonstrated increased blood glucose and GSP levels reflecting their diabetic state compared to SD controls (Figure 1B,C). ZDSD blood glucose levels continued to increase throughout this experiment, consistent with published reports that demonstrate increasing blood glucose levels throughout the ZDSD rat lifetime [31]. GSP was measured due to its superior ability to monitor metabolic alterations over a short time period compared to HbA1c, which measures average blood glucose over several months [37]. While no difference in glucose was detected between the ZDSD injured and ZDSD injured + PM groups, GSP levels were significantly lower in the ZDSD injured + PM group than in the ZDSD injured group. Interestingly, serum levels of GSP (fructosamine) measured in standard SD rats are much lower (<20 μM) than those measured in humans, in whom ≤ 285 μM of fructosamine is considered normal [38]. Whether these results are assay specific or rats generally have lower GSP levels should be investigated in the future. There were no significant differences in daily water uptake in ZDSD rats after IVD injury, regardless of whether PM had been added to the water (Figure 1D). This is important to note, since the PM treatment was given in the drinking water and the dose was calculated based on each rat’s daily water consumption.

T2-weighted µMRI analysis demonstrated a reduction in NP hydration at 8 weeks post-surgery compared to pre-surgical and uninjured IVDs in both the ZDSD and SD groups (Figure 2). This successful induction of IVD degeneration in previously healthy SD rats using an 18G needle was similar to that done in previous studies by our group and others using the same animal model [12,39]. Treatment with PM tended to attenuate the NP dehydration; however, no significant differences were detected between the ZDSD injured and ZDSD injured + PM groups (Figure 2B). To our knowledge, no prior study has investigated the effect of PM on IVD degeneration using µMRI. Furthermore, our results indicated no differences in NP hydration between SD and ZDSD rats in response to IVD injury. In contrast to our findings, differences in µMRI values between diabetic (streptozotocin [STZ] induced) and non-diabetic SD rats were detected in a recent study by Zhang et al. [40]. Those authors performed disc injury on caudal discs using a 27G needle and detected differences at week 4 post-surgery. In a clinical study, differences in IVD degeneration were detected between diabetic and non-diabetic twins using MRI [41]. However, this association between T2DM and intervertebral disc disease was dependent on the body mass index. Comparing our findings with those in the literature [40,41], we find that a less severe degeneration model, earlier time points of MRI measurement, and a model with comparable weight as the control animal should be considered to detect differences in IVD hydration using MRI in the future. In addition, the use of T2* or T1rho sequences, known to be correlative to the glycosaminoglycan content of collagen content in humans, would be valuable. Once these sequences are available for smaller scanners that can be used for rodents, their use should be considered to measure more specific differences in the various experimental groups [42,43].

Immunohistochemical staining using an antibody known to detect a wide range of AGEs showed higher AGE levels in the NP and in the endplate cartilage in the uninjured IVDs of ZDSD versus SD rats (Figure 5). However, an investigation of the protein expression levels of AGE compounds (CML, CEL, and pentosidine), using ELISA, demonstrated no differences in AGE levels between uninjured IVDs in SD and ZDSD rats (Figure 4A). Similar to our immunohistochemical data observations, Fields at al. reported a significant increase in AGE in the IVDs of a T2DM rat model compared to SD rats [44]. We speculate that the observed differences in the IVD tissue–related results of the two AGE assays (IHC vs. ELISA) are due to differences in the compound specificities of the two different antibodies that were used [16,17,18].

Surprisingly, a similar increase in AGE levels was detected in ZDSD versus control SD rats following IVD injury (Figure 4A and Figure 5). These findings indicate that AGE accumulation may accompany acute IVD degeneration in addition to its known accumulation in diabetes. Previous research describes the formation of AGEs as a significant abnormality that occurs in diabetes mellitus and, likely, in inflammation [45], which is a critical step in the progression of IVD degeneration. Since the weight of our SD rats at study end was comparable to obese CD IGS SD rats at 28 weeks of age [36], we cannot exclude that the loading conditions on the disc or undiagnosed obesity contributed to our findings on AGE regulation in the SD model. We also detected a decrease in aggrecan levels in injured versus uninjured IVDs in SD rats (Figure 4B). The destructive effect of AGEs on IVD extracellular matrix proteins was recently reported by Hoy et al. [46]. In their study, the authors demonstrated a receptor for AGE (RAGE)—dependent AF collagen disruption in mice fed a high-dose AGE diet, which they suggest induced early degenerative changes in the disc [46]. Therefore, the reduced aggrecan levels detected in our study might have been a consequence of the increase in AGE compounds detected in the degenerated IVD.

The mechanism by which AGEs may enhance IVD degeneration in diabetes has been described in prior studies, which suggest that AGE accumulation as a result of hyperglycemia may impair the biological properties of the disc [44,47]. For example, Sivan et al. hypothesized that AGEs accumulate in major proteins, including in aggrecan and collagen, leading to further alterations in NP cell biology and thus preventing extracellular matrix repair and turnover [22]. From the detection of increased AGE levels in degenerated IVDs of both diabetic and non-diabetic rats in this study, we infer that the elevated AGE levels are potentially consequences of both systemic hyperglycemia and changes in the local environment of the IVD.

A decrease in AGE levels was detected in the injured IVDs of animals receiving PM treatment versus animals receiving water only, as demonstrated by ELISA and IHC. While the ability of PM to inhibit the formation of AGEs in both STZ-induced diabetic and Zucker (obese, hyperlipidemic) rats has been described [48,49], the potential of PM to reduce IVD degeneration in a rat model of T2DM has not previously been reported. In mice with STZ-induced diabetes, anti-AGE oral treatment in combination with the broad-acting anti-inflammatory, pentosan polysulfate, has been shown to prevent or reduce degenerative changes in IVDs [25]. Therefore, it is likely that PM hinders AGE accumulation in the IVD, which may protect NP cells and the surrounding matrix from pathological changes, and thus attenuates the degeneration-induced destruction of disc tissue. Indeed, our results detected higher aggrecan levels in PM-treated animals than in untreated controls (Figure 4B). Further research is needed to elucidate the protective mechanisms of PM in IVD degeneration in diabetic and non-diabetic individuals.

Our study was not without limitations. 1. Only male rats were included in the study due to reports that ZDSD males have a more consistent pattern of becoming diabetic [31]. This may have caused sex-biased results since outcomes with female rats could have been different. In future research, we may consider other models to study IVD degeneration in diabetes in male and female animals. 2. Our sample numbers differed between assays and groups. In cases where no statistical significance was detected, a higher number of samples may have resulted in significant differences. 3. The effect of PM on IVD degeneration was investigated in ZDSD rats, but not in SD rats, since the focus of this study was on the effect of PM treatment in diabetic conditions. However, in light of the fact that our results show increased AGE levels in the IVDs of both rat types, the effect of PM on disc degeneration in control SD rats (both normal and obese) should be investigated in future studies. 4. The effect of PM on the attenuation of disc dehydration, as detected by µMRI, was limited. Our injury model induced severe disc degeneration [12]. A more moderate IVD degeneration model might be advised for future PM testing, or PM could be applied in combination with additional treatments, such as local stem cell injections, allowing for the reduction of AGE-induced processes while re-populating the IVD with healthy NP cells to replace those cells that may have become apoptotic during IVD degeneration.

## 4. Materials and Methods

### 4.1. Study Design

Male Zucker Diabetic Sprague-Dawley (ZDSD) rats (Crown Biosciences, San Diego, CA, USA) were chosen because they show a T2DM progression similar to that of the human disease, with pre-diabetes at 8–16 weeks of age, overt diabetes at 16+ weeks of age, and diabetic complications at 24+ weeks of age [32]. Based on the literature, ZDSD males have a more consistent pattern of becoming diabetic [31]. To limit variability between animals as much as possible, we thus focused on male rats only. Prediabetic ZDSD rats were synchronized to a diabetic phenotype by 3 weeks of a high-fat diet (Purina 5SCA/TestDiet^®^ Rodent Diet 5SCA, LabDiet) beginning at age 16 weeks by the vendor (Crown Biosciences, San Diego, CA, USA). The rats were then shipped to our animal facility and switched to a high-energy diet (Purina Rodent LabDiet 5008, 27% protein, 17% fat, 57% carbohydrates) for the whole study duration to maintain the diabetic phenotype. At 20 weeks of age, the rats underwent anterior lumbar disc surgery in accordance with the IACUC protocol (Cedars-Sinai Medical Center, Identifying and mitigating the effects of diabetes on disc degeneration, #IACUC008089, approval date: 1 May 2019–30 April 2021). Eighteen ZDSD and 14 healthy, male CD^®^ Sprague Dawley IGS (SD) rats (Charles River, Wilmington, MA, USA), both 20 weeks of age, were included in the study. Caesarean Derived (CD) Sprague Dawley rats were used as the control, since SD rats are a widely used outbred model for the investigation of IVD degeneration [50,51]. The advantage of SD rats is that their spines are relatively large and therefore easier to handle surgically. Another reason for choosing the SD rat model as the control in our study is the fact that the ZDSD rat has been derived by crossing a sub-strain of the SD rat with a homozygous lean Zucker Diabetic Fatty rat (ZDFfa/+). Compared to the SAS SD sub-strain, the CD International Genetic Standard (IGS) SD rat model weighs on average 15% more at 15 weeks of age, according to the vendor’s website (Charles River, MA, USA). It has been demonstrated that obesity can be induced by diet in the CD IGS SD model [36]. In the present study, SD rats were fed regular rat chow (PicoLab Rodent Diet 20, 20% protein, 10% fat, 62% carbohydrates) throughout the duration of the study. Prior to surgery and at euthanasia, the rats were weighed, and their diabetic condition was evaluated by performing blood glucose tests (True Metrix, CVS Health). SD and ZDSD rats were randomly assigned to 2 different surgical groups: (1) IVD injury created with an 18G needle as previously reported [12] and (2) sham surgery only. ZDSD rats with injured IVDs were randomly assigned to a PM treatment group or a water control group. PM was given per os at a daily dose of 200 mg/kg/day, which has previously been reported to be efficient in mice [26]. PM uptake was monitored and adjusted on a daily basis, starting at day 1 post-surgery until the animals were sacrificed at week 8. The number of animals used in the different groups was at follows: SD uninjured: *n* = 6, SD injured: *n* = 8, ZDSD uninjured: *n* = 5, ZDSD injured: *n* = 8, ZDSD injured + PM: *n* = 5. To investigate blood glucose levels and the progression of IVD degeneration in the various experimental groups, the following methods were employed: pre-surgery and at 8-weeks post-surgery, T1- and T2-weighted µMRI analyses of spinal segments were performed; and at 8-weeks post-surgery, glycated serum protein (GSP) analysis of blood samples, protein analysis of the IVDs via ELISA, histology, and immunohistochemical staining were conducted. Sample exclusion criteria in the assay analysis were a poor scan quality for MRI images and blood contamination for serum samples.

### 4.2. Surgical Approach

Under inhalation anesthesia and after incision, an 18G needle was inserted (using an anterior approach) into lumbar IVDs L3-4, L4-5 and L5-6 under direct visual and fluoroscopic guidance, as previously described in detail [12]. An 18G needle was chosen because a recent study by our group demonstrated this needle size to induce reproducible, lumber IVD degeneration that resulted in hypersensitivity and analgesia [12]. Fluoroscopic guidance was used to ensure precise needle insertion [52]. Pain medication (0.05 mg/kg buprenorphine, subcutanous) was administered at the time of surgery and 12 h after surgery. Antibiotics (5 mg/kg Baytril, intraperitoneal) were given on days 1–3 post-surgery to prevent infections. Rats were single housed after surgery to minimize the risk of injury from companions. The rats’ welfare was assessed daily until sacrifice at week 8 post-surgery.

### 4.3. Micro-Magnetic Resonance Imaging

To visualize the IVD structure and level of hydration, µMRI imaging was employed using a small animal magnetic resonance imaging scanner, Bruker BioSpec 9.4T (94/20) with Avance III electronics 9.4T, as previously described by our group [12,53]. While the animal was given inhalation anesthesia, μMRI was performed at the Imaging Core facility under the Imaging Core’s approved IACUC protocol (Cedars-Sinai Medical Center, Core Protocol: Optical Imaging for Rat Research, #IACUC006110, approval date: 1 May 2019–30 April 2021)). Utilizing the iliac crest as the anatomical landmark for each scan, regions of interest (ROIs) of IVDs L3-4, L4-5, and L5-6 were manually contoured by an independent clinician researcher who was blinded to the conditions for measurements.

### 4.4. Glycated Serum Protein and Enzyme-Linked Immunosorbent Assays

Serum was derived from blood, obtained pre-surgery and at 8 weeks post-surgery, from the tail of each rat. For glycated serum quantification, the Rat Glycated Serum Proteins (GSP) Assay Kit (Crystal Chem, Elk Grove Village, IL, USA) was employed according to the manufacturer’s instructions. The kit uses proteinases to digest serum proteins into low-molecular-weight glycated protein fragments, and uses specific fructosaminase to yield glucosone and H_2_O_2_, which is then measured by a colorimetric reaction. The absorbance at 570 nm is proportional to the concentration of GSP. Each sample was analyzed in duplicates. IVDs were isolated from spine segments L3-4, 4-5, and L5-6, and IVDs were stored at −80 °C. For protein extraction, each sample was homogenized with scissors in RIPA lysis buffer supplemented with Halt protease inhibitor cocktail (Thermo Scientific Scientific, West Hills, CA, USA). After a 15-min incubation on ice, samples were centrifuged at 13,000× *g* for 15 min and the protein containing supernatant collected. ELISA kits for rat AGE (detecting AGE compounds CML, CEL, and pentosidine, Mybiosource, SanDiego, CA, USA) and aggrecan (Quantikine ELISA, R&D systems, Minneapolis, MN, USA) were employed to detect protein levels in each IVD. Results were normalized to total protein levels, which were determined by a BCA protein assay, performed according to manufacturer’s protocol (Thermo Fisher Scientific, West Hills, CA, USA).

### 4.5. Histology and Immunohistochemistry

Spine sections were prepared for histological analysis as described in our prior study [11]. After decalcification for 30 days in EDTA, the spines were cut across the vertebral bodies from lumbar spine L3 down to L6, resulting in single vertebral segments (vertebra-disc-vertebra). Vertebral segments were dissected in the mid-sagittal plane, processed for paraffin embedding, and sectioned to a thickness of 5 μm. The sections were stained with hematoxylin and eosin (H&E) or Alcian blue/Picrosirius red, containing Alcian blue staining solution (pH 2.5) in 0.5% aqueous acetic acid (Sigma, Burbank, CA, USA), and Picrosirius red staining solution (0.1 g Sirius red/100 mL picric acid). For AGE staining, slides were deparaffinized with xylene and hydrated using graded alcohol. A 20-min Proteinase K (Agilent Technologies, US, S3020, USA) digestion was used to unmask antigens. Slides were incubated overnight with Rabbit anti-AGE polyclonal antibody (1:200 dilution, Abcam, Cambridge, MA, USA, ab23722). Next, ImmPRESS^®^ Excel Amplified Polymer Staining Kit Anti-Rabbit IgG Peroxidase (Vector Laboratories, Burlingame, CA, USA, MP-7601) was used where slides were incubated with ImmPACT DAB EqV reagents for 4 min and counterstained with Hematoxylin QS (Vector Laboratories, Burlingame, CA, USA, H-3404-100) for 45 s. Finally, slides were dehydrated and coverslipped with Permount mounting medium (Fisher Scientific, Waltham, MA, USA, SP15-100). Stained slides were scanned with an Aperio R slide scanner (Leica Microsystems, Buffalo Grove, IL, USA) and analyzed for evidence of changes in the NP and AF using QuPath Bioimaging Software v0.2.0. The morphological features that were evaluated included lesion of the NP, the sharpness of the boundary between the NP and the AF, and disruption of the AF. Histological and immunohistochemistry results were evaluated by two independent interpreters.

### 4.6. Statistics

All statistical analyses were performed using Prism 8 (GraphPad Software, Inc., La Jolla, CA, USA); *p* < 0.05 was considered to be statistically significant. There were 3 outcome measurements: (1) levels of blood glycation, (2) µMRI measures, and (3) levels of protein expression. To minimize inter-animal variability, changes in normalized results with time within and across experimental groups were analyzed for outcomes measures 2 and 3. Separately for each dependent outcome measure, analysis of variance (two-way ANOVA) was performed. Mean values were compared across experimental groups; for multiple comparisons, appropriate post hoc tests were used. In the figures, results are presented as violin plots with median values and individual data points are shown.

## 5. Conclusions

Using a type 2 diabetes mellitus—IVD degeneration rat model, this study demonstrates the potential of oral PM treatment to reduce levels of GSPs and to attenuate AGE levels and NP destruction in injured IVDs in diabetes. Therefore, PM may be considered a non-invasive biologic treatment to mitigate the risk of developing degenerative disc disease specifically in diabetic patients. Interestingly, while increased levels of AGE compounds were detected in the uninjured IVDs and endplates of ZDSD rats compared to SD rats, IVD injury resulted in a significant AGE accumulation in both ZDSD and SD rats. This observation may be a result of an undiagnosed obesity in the SD rats and/or a local response to IVD injury. Therefore, the regulation of AGE levels and the effect of PM on disc degeneration in SD rats with and without obesity should be considered in future research.

## Abbreviations

T2DM	Type 2 Diabetes Mellitus
AGE	advanced glycation end products
IVD	intervertebral disc
NP	nucleus pulposus
AF	annulus fibrosus
PM	pyridoxamine
ZDSD	Zucker Diabetic Sprague Dawley rat
SD	Sprague Dawley rat
MRI	magnetic resonance imaging
LBP	low back pain
CML	Nε-(carboxymethyl)lysine
CEL	Nε-(carboxyethyl)lysine
ZDFfa/+	Zucker Diabetic Fatty rats
G	needle gauge
L	lumbar level
ROI	region of interest
IACUC	institutional animal care and use committee
ELISA	enzyme-linked immunosorbent assay
BCA	bicinchoninic acid assay
EDTA	ethylenediaminetetraacetic acid
H&E	hematoxylin and eosin
GSP	glycated serum protein
HbA1c	hemoglobin A1c
STZ	streptozotocin
RAGE	receptor for advanced glycation endproducts
IHC	Immunohistochemistry
NPCs	nucleus pulposus cells
vs	versus
